# Caries in a cohort of adults with cystic fibrosis: a cross-sectional study

**DOI:** 10.1038/s41415-024-8269-8

**Published:** 2025-04-25

**Authors:** Fiona O´Leary, Niamh Coffey, Francis M. Burke, Anthony Roberts, Laura Kirwan, Paul O´Regan, Barry Plant, Martina Hayes

**Affiliations:** 41415349131001 Clinical Fellow in Restorative Dentistry, Cork University Dental School and Hospital, Wilton, Cork, Ireland; 41415349131002 Consultant in Restorative Dentistry, Cork University Dental School and Hospital, Wilton, Cork, Ireland; 41415349131003 PhD, Cystic Fibrosis Registry of Ireland, Dublin, Ireland; 41415349131004 Cystic Fibrosis Registry of Ireland, Dublin, Ireland; 41415349131005 Consultant Respiratory Physician, Cork University Hospital, Wilton, Cork, Ireland; 41415349131006 Associate Professor and Consultant in Restorative Dentistry, Dublin Dental University Hospital, Dublin, Ireland

## Abstract

**Objectives** To measure past dental caries experience in people with cystic fibrosis and to compare the results with a control group of people without cystic fibrosis.

**Methods** A cross-sectional study of 92 adults with cystic fibrosis and 92 adults without cystic fibrosis was undertaken in Cork University Dental School and Hospital. The median age for study group and control group participants was 31 years and 27 years, respectively. All participants completed a detailed questionnaire before undergoing a clinical examination that recorded demographic, social and oral health variables. Caries was recorded using the Decayed, Missing and Filled Teeth (DMFT) index. All data were statistically analysed using the Wilcoxon rank-sum test, chi-squared test and Fisher's test. Negative binomial models were also used to analyse data.

**Results** The study group had a higher mean DMFT score compared to the control group (6.52, 0.99, 0.41, 3.89 versus 5.33, 0.18, 0.11, 3.68). While the study group had a higher DMFT, the only component that was statistically significant between the groups was the Decayed Teeth component (p <0.001).

**Conclusion** In this study, the cohort of people with cystic fibrosis had more caries than people without cystic fibrosis. Further research is required to establish if underlying systemic conditions, social and behavioural factors, or a combination of the aforementioned are responsible for a higher caries experience in this study group.

## Key points

- This paper reports that adults with cystic fibrosis have a higher caries experience than people without cystic fibrosis.
- It discusses caries risk factors that are specific to people with cystic fibrosis.
- It highlights the need for preventive dental health policy for this cohort of medically compromised people.

## Introduction

The oral health of people with cystic fibrosis (PwCF) is an area that is under-researched, possibly as a consequence of historic premature mortality and systemic manifestations of the condition taking precedent. However, with the advent of new pharmacological therapies, notably gene modulators that target the underlying genetic mutation, people are now living longer with the disease^1^ and new health concerns that were previously not of concern are arising in an older population.^2^ One such concern is the area of oral health. This concern was expressed by patient advocates from Cystic Fibrosis Ireland (CFI). This is a voluntary organisation that was established by parents in 1963 to improve the treatment and facilities for PwCF in Ireland. CFI liaises with medical professionals to give assistance to parents, families, carers and PwCF with CF and advocates to improve the lives of those with CF.^3^ Dental caries is a prevalent chronic infectious disease resulting from tooth-adherent cariogenic bacteria that metabolise carbohydrates to produce acid, which over time leads to the dissolution of tooth mineral (primarily hydroxyapatite).^4^ This can lead to cavities forming in the tooth which, if left untreated, can cause pain, infection, aesthetic concerns and even tooth loss.^5^ Untreated dental caries in permanent teeth is the most prevalent disease globally.^6^

Previous studies assessing oral health in PwCF have identified caries risk factors unique to this population. These include higher levels of cariogenic (dental caries-causing) bacteria *Streptococcus mutans,* gastroesophageal reflux disease (GERD), a high-sugar diet and habitual grazing, reduced or altered salivary function, and a higher prevalence of enamel defects.^7^^,^^8^^,^^9^ The vast majority of previous studies assessing oral health have primarily focused on paediatric or adolescent populations; adults with the disease account for only a small proportion of study subjects.^8^^,^^10^^,^^11^^,^^12^^,^^13^ With this in mind, it is timely that current research and future research examines adults with the CF as oral pathologies (dental caries and periodontal disease) are more prevalent in older populations. The majority of oral diseases are largely preventable. The importance of identifying populations that may have a higher risk and/or higher levels of dental disease cannot be undervalued.^14^ Good oral hygiene and a disease-free dentition are particularly important for PwCF when one considers that teeth may serve as a reservoir for respiratory pathogen colonisation.^15^ PwCF are often considered for solid organ transplantation and are often placed on bone-targeted therapies at a young age. Dental disease may delay treatment where solid organ transplantation is required or where bone-targeted therapies are required. As a prerequisite, certification of oral health from a dental practitioner is required. This is necessary to minimise the risk of postoperative infection arising from oral pathology^16^ and medicine-related osteonecrosis of the jaw should surgical dental treatment be required, respectively.^17^ It is timely that the oral health of adults with CF is examined to identify if this group has higher levels of dental caries. Identifying high-risk populations allows policymakers to selectively target these groups for preventive dental services, improving health outcomes, and the effectiveness of services. The objective of this cross-sectional study was to measure the dental caries experience in a cohort of PwCF and compare the results with a control group of people without CF.

## Materials and methods

### Ethical approval

The study design was submitted to and given full ethical approval by the Clinical Ethics Committee of the Cork Teaching Hospitals (ECM 03/2022 PUB). The study was conducted in compliance with the principles of the Declaration of Helsinki and written informed consent was obtained from each participant.

### Recruitment

All adults (≥18 years) who were under the care of the adult CF unit in Cork University Hospital (n *=* 180) were invited to undergo a dental examination. The final number recruited for the study group was n *=* 92. A population-based control group of adults (≥18 years) was recruited (n *=* 92). Control participants were invited to attend Cork Dental School and Hospital for a free dental examination through adverts placed in local shopping centres, community centres and on social media channels related to the study. Telephone and email contact details of the study coordinator were provided, and patients were allocated appointments following correspondence with the study coordinator, provided they met the inclusion criteria. The study coordinator confirmed that all control participants had a negative CF diagnosis, no familial history of CF, and were systemically healthy and not taking long-term medication. No financial rewards were offered for study participation. Data were collected between September 2020 and October 2022. The study conforms to the Strengthening the Reporting of Observational Studies in Epidemiology (STROBE) guidelines for reporting cross-sectional studies.^18^

### Inclusion and exclusion criteria

The inclusion criteria for entering the study group:

- Individuals aged 18 years and above with a positive CF diagnosis as confirmed by previous sweat and genetic tests
- Individuals living in Ireland.

The inclusion criteria for entering the control group:

- Individuals aged 18 years and above with a negative CF diagnosis
- Individuals living in Ireland
- Systemically healthy with no long-term medication.

The exclusion criteria for this study:

- People with a familial history of CF.

## Data collection and oral examination

### Data collection

Before undergoing a clinical examination, all participants completed the World Health Organization's (WHO's) ‘Oral health questionnaire for adults' with assistance from a research assistant. This questionnaire records data related to demographic, social and behavioural variables. Additional questions were included specifically for PwCF following input from patient advocates from CFI. These questions recorded information pertaining to CF pharmacological therapies, such as pancreatic enzyme replacement therapy (PERT), oral nutritional supplements (ONS), azithromycin (AZT) and pulmonary status measured by forced expiratory volume (ppFEV1).

### Examiner calibration

Oral examinations were conducted by two examiners (FOL and NC). Both examiners underwent training and calibration for caries detection and measurement. Examiners were provided with a manual outlining the study protocols and caries examination criteria. Both examiners conducted caries examinations using the Decayed, Missing, Filled Teeth index (DMFT) on individuals attending the restorative clinic in Cork University Dental School and Hospital. These examinations were overseen and calibrated for caries detection against a gold standard examiner. Inter-examiner agreement was tested against a gold standard examiner. The inter-examiner reproducibility was measured as 0.95 by the kappa statistic, indicating a high inter-rater reliability.

### Oral examination

All examinations were conducted using a plane surface mouth mirror (mirror head size 4, front), a WHO community periodontal index probe. The clinical examination was conducted following the recommendations outlined for oral epidemiological studies by the WHO. Study group participants (PwCF) were examined within a single examination room in the adult CF unit in Cork University Hospital using a Daray lamp light.

Control participants (people without CF) were examined in a standard dental operatory in Cork Dental School and Hospital. This was a visual examination with no radiographic examinations. The WHO community periodontal index probe was used if necessary to confirm the presence of caries. Caries experience was measured using the WHO-approved caries measurement index DMFT, which measured carious (Decayed) Teeth (DT), Missing Teeth (MT), and Filled Teeth (FT). As per the guidelines, the status of permanent teeth were recorded as a score from 0-9. Score descriptors are outlined in Table 1. As per guidelines, the DT component includes all teeth with codes 1 or 2. The stages of caries that precede cavitation were excluded because they could not be reliably identified in field conditions. Therefore, teeth were given a score of 1 or 2 when a lesion in a pit or fissure, or on a smooth tooth surface, had an unmistakable cavity, undermined enamel, or a detectably softened floor or wall when probed. The MT component comprised of teeth coded 4 in subjects under 30 years of age, and teeth coded 4 or 5 in subjects 30 years and older ie missing tooth due to caries or for any other reason. The FT component included only teeth with code 3. Teeth coded 6 (fissure sealant) or 7 (fixed dental prosthesis/bridge abutment, special crown or veneer/implant) were not included in calculations of DMFT. DMFT is the sum of the number of carious teeth (DT), missing due to caries (MT), and filled teeth (FT) in the permanent dentition. All permanent teeth except for third molars (wisdom teeth) were examined. So, for an adult with a full permanent dentition, DMFT ranges from 0-28. The higher the DMFT value, the higher the experience of untreated disease, and previous dental treatment received by an individual to date.

**Table 1:** Codes for DMFT measurement^19^

**Score**	**Description**
0	Healthy
1	Caries
2	Filled with caries
3	Filled no caries
4	Missing due to caries
5	Missing for another reason
6	Fissure sealant
7	Fixed partial denture/crown, abutment, veneer, implant
8	Unerupted
9	Not recorded

### Statistical analysis

Study participant characteristics collected in the questionnaire were summarised using median (interquartile range [IQR]) for continuous variables, and frequency (percentage) for categorical variables. The case group and control group were compared using Wilcoxon rank-sum tests, chi-squared tests or Fisher's exact tests where frequencies were less than five.

Teeth were assessed for DT, MT and FT, described as the DMFT indicator, and the mean DMFT for both the CF and the control group was calculated. Mean DMFT were compared using Wilcoxon rank-sum tests.

A binomial model was fitted for caries (yes/no as defined by a score of 1 and 2), including the effects of CF status and sex. The effects of sex were analysed to determine if women in this study had higher caries experience as per trends in the general population.^20^ Three negative binomial models were fitted for DMFT total and the following variables: Model 1 = DMFT including the effects of CF status and sex; Model 2 = DMFT total including the effects of CF status, sex, education, diet, sugary drinks and dry mouth; and Model 3 = DMFT total, including the effects of CF status, sex, toothpaste, toothbrushing, last dental attendance and water fluoridation.

A binomial model was fitted for DMFT and variables specific to CF: PERT, AZT, ONS, and lung function, measured by the percent predicted forced expiratory volume in one second (ppFEV1).

An alpha level of 5% was used for all tests to determine when the difference was statistically significant (p <0.05). R version (4.2.0) was used for all statistical analysis.

## Results

### Characteristics of participants

In total, 92 PwCF were included in the study group; 52 (n = 57%) men with CF and 40 women (n = 43%) with CF were recruited. Additionally, 49 (n = 53%) women and 43 (n = 47%) men were recruited for the control group. The median age for study group participants was 31 years (IQR: 25.0-35.8). The median age for control group participants was slightly younger at 27 years (IQR: 23.0-33.0). Demographic, social and education characteristics of participants are outlined in Table 2. While there was no statistical difference between the study and control groups in sex, education, smoking and alcohol use, there was a difference in the median age (p *=* 0.044), with the CF group having a higher median age.

**Table 2:** Characteristics of study participants. Median and IQR are presented for continuous variables, and the case and control groups are compared using the Wilcoxon rank sum test. Frequency and percentage are presented for categorical variables, and the CF and non-CF groups are compared using chi-squared tests (or Fisher's exact test where frequencies are less than five). The same table has been used in another paper looking at the prevalence of enamel defects in the same cohort^9^

		**CF (n = 92)**	**Non-CF (n = 92)**	**P-value**
Sex	Male	52 (56.5%)	43 (46.7%)	0.238
	Female	40 (43.5%)	49 (53.3%)	
Median age in years (IQR)		31 (25.0-35.8)	27 (23.0-33.0)	0.044
Education	Primary	1 (1.2%)	2 (2.2%)	
	During secondary	2 (2.3%)	2 (2.2%)	
	Secondary	25 (29.1%)	23 (25%)	
	Third level	58 (67.4%)	65 (70.7%)	0.889
Smoking		1 (1.1%)	7 (7.6%)	0.078
Alcohol use		67 (76.1%)	68 (74.7%)	0.964
Diet and dietary habits	3 or less meals per day	10 (11.2%)	36 (39.1%)	
	4 or 5 meals per day	59 (66.3%)	49 (53.3%)	
	6 or 7 meals per day	17 (19.1%)	6 (6.5%)	
	More than 7 meals per day	3 (3.4%)	1 (1.1%)	<0.001
Sugary drinks	None	5 (5.6%)	19 (20.7%)	
	Once a day	26 (29.2%)	45 (48.9%)	
	Twice a day	28 (31.5%)	22 (23.9%)	
	3 times a day	14 (15.7%)	5 (5.4%)	
	4 times a day or more	17 (18.9%)	1 (1.1%)	<0.001
How do your teeth affect your ability to chew your food	Not at all	81 (92.0)%	84 (91.3%)	
	Somewhat affects me	7 (8.0)%	6 (6.5%)	
	Affects me alot	0 (0%)	2 (2.2%)	0.5663
Fluoride exposure	Toothpaste has fluoride	71 (79.8%)	88 (95.7%)	
	Toothpaste has no fluoride	0 (0%)	2 (2.2%)	
	Unsure	18 (20.2%)	2 (2.2%)	<0.001
Toothbrushing	Twice a day or more	67 (72.3%)	85 (92.4%)	
	Once a day or less	22 (24.7%)	7 (7.6%)	0.003
Use of dental service	Has own GDP? Yes	73(79.8%)	69(75%)	0.555
	Has own GDP? No	19(20.2%)	23(25%)	
Reason for seeking dental services	Checkup at least once a year	44(48.3%)	53(57.6%)	0.029
	Checkup at least twice a year	7(7.9%)	14(15.2%)	
	When treatment is needed	14(15.7%)	15(16.3%)	
	Pain/ problem	16(16.9%)	8(8.7%)	
	Never attend the dentist	10(11.2%)	2(2.2%)	
Aesthetic concerns	Does not affect me	38 (42.7%)	54 (58.7%)	
	Affects me	51 (57.3%)	38 (41.3%)	0.0451
Medication	Osteoporosis	9 (10.2%)	0 (0%)	
	Osteopenia	8 (9.1%)	6 (6.5%)	
	No	71 (80.7%)	86 (93.5%)	0.002
	Bisphosphonates	10 (11.6%)	0 (0%)	<0.001
	Oral nutritional supplements	17 (19.1%)	1 (1.1%)	<0.001
	Antibiotics	1 (1.3%)	0(0%)	0.918

### Oral health parameters

When oral health parameters were analysed, the CF group brushed less frequently than the non-CF group (p = 0.003). PwCF attended the dentist less frequently for check-ups (p *=* 0.029), with a higher number of these individuals attending when they needed treatment or were in pain. PwCF reported more aesthetic concerns than non-CF people (p *=* 0.045). Within this study, 34.1% (n = 63) of people in the study group had a public water supply whereas 78.3% (n = 72) of people in the control group had a public water supply. Therefore, more people without CF had access to water fluoridation.

### Oral health status

The percentage of PwCF who had untreated dental caries present (DT) (DMFT code 1 and/or 2) during the clinical examination was 38% compared to just 13% of people in the control group. A binomial model was fitted for caries status (yes/no) with the variable of CF status and the p-value for this variable significant (p *=* 0*.*0002). When the variable of sex was added to this model, the inclusion of sex was significant (p *=* 0.0225).

Female participants in both groups had more untreated dental caries than male participants, which is summarised in Table 3. The percentage of study participants and control participants with missing teeth (MT) (DMFT code 4 in subjects under 30 years of age, and 4 or 5 in subjects 30 years and older) was 15.2% and 7.6%, respectively. In total, 76% of people with CF had FT and 73.9% of people in the control group had FT.

**Table 3:** Percentage of individuals with caries present (DMFT code 1 and/or 2) based on CF status and sex

	**Caries present (%)**	**No caries (%)**
Male CF (n = 52)	26.9%	73.1%
Female CF (n = 40)	52.5%	47.5%
Male non-CF (n = 43)	11.6%	88.4%
Female non-CF (n = 49)	14.3%	85.7%

The mean DMFT were calculated for both the study and control groups. These are presented in Table 4. Among these, the mean values were higher in the study group compared to the control group but only DT were statistically significantly higher (p <0.001). There were more untreated DT in the CF group than the control group (0.99 versus 0.18). No significant differences were found in the FT (3.89 versus 3.68) and MT (0.41 versus 0.11) components between the two groups.

**Table 4:** Mean DMFT, DT, MT, FT with Wilcoxon p-values, SD and IQR for the CF and non-CF groups

	**CF group** **Mean (SD)**	**Non-CF group** **Mean (SD)**	**Wilcoxon p-value**	**CF group** **Median (IQR)**	**Non-CF** **Median (IQR)**
DMFT	6.5 (5.0)	5.3 (3.9)	0.14	6 (2.8−10)	5 (2−7)
DT	0.99 (1.8)	0.2 (0.55)	<0.001	0 (0−1)	0 (0−0)
MT	0.41 (1.7)	0.1 (0.48)	0.09	0 (0−0)	0 (0−0)
FT	3.9 (3.9)	3.7 (3.6)	0.81	3 (0−6.25)	3 (0−5.1)

### DMFT total

Three negative binomial models were fitted for DMFT total. Table 5 summarises the output from these. Model 1 shows that the incident rate of DMFT for people with CF is 1.3 times higher than that of the control group. When the variable of sex is analysed, women have a 1.3 times higher incident rate than men. Model 2 shows incidence when demographic and behavioural variable are included. The incidence rate of DMFT was lower for individuals across both groups with secondary and third level education. As expected, the incidence was higher with increased meal intake per day (four or five meals per day [1.4]). Model 3 looks at dental health parameters of toothpaste, toothbrushing, dental attendance and water fluoridation status, as well as any significant demographic variables. Individuals who brush less than once a day have a higher DMFT (1.8) than those who brush once a day (1.14).

**Table 5:** Negative Binomial regression analysis of CF associated with total DMFT for three models

**Total number of teeth with DMT prevalence ratio (95% CI)**	**Variables**	**Model 1: Baseline**	**Model 2: Baseline + demographic and behavioural variables**	**Model 3: Model 2 + dental health factors**
Intercept		4.5 (3.7-5.6)***	3.6 (1.7-8.0)***	5.6 (4.1-7.8)***
CF status		1.2 (0.99-1.5)	1.2 (0.93-1.5)	1.2 (1.0-1.6)*
Sex	Female	1.3 (1.0-1.6)*	1.3 (1.0-1.6)*	1.3 (1.0-1.7)**
Education level	Secondary level		0.71 (0.35-1.3)	
	Third level		0.76 (0.38-1.4)	
Diet and dietary habits	Four or five meals per day		1.3 (1.0-1.7)*	
	Six or seven meals per day		1.4 (0.97-2.2)	
	More than seven meals per day		1.7 (0.81-3.9)	
Sugary drinks	Once a day		1.4 (0.95-2.0)	
	Twice a day		1.46 (0.98-2.1)	
	Three times a day		1.4 (0.98-2.1)	
	Four times a day or more		1.1 (0.64-1.8)	
Dry mouth	No		1.0 (0.81-1.3)	
Fluoride exposure	Toothpaste has no fluoride			0.60 (0.19-1.9)
	Unsure			0.76 (0.51-1.1)
Toothbrushing	Once a day			1.1 (0.81-1.6)
	Less than once a day			1.8 (1.1-3.2)*
Last attendance	Less than one year ago			0.76 (0.56-1.0)
	Less than two years ago			0.95 (0.67-1.3)
	Less than three years ago			0.86 (0.56-1.3)
	More than three years			0.72 (0.52-0.99)*
Water fluoridation	Public			0.88 (0.66-1.1)
	Unsure			0.49 (0.28-0.89)*
Key: *p <0.05 **p <0.01 ***p <0.001 Note = Model 1: CF status and sex as the independent variables; Model 2: Model 1 plus demographic variables; Model 3: significant predictors from Model 2 plus health factors				

### DMFT total with cystic fibrosis specific variables

Two negative binomial models were fitted for DMFT total and variables specific to PwCF: PERT, AZT, ONS and ppFEV1. Debris total was also included in these models which are summarised in Table 6. There were no significant differences within these models; however, people taking AZT and those with a higher debris total had a higher DMFT (1.3 and 1.0, respectively).

**Table 6:** Negative binomial regression analysis of CF specific variables associated with total DMFT for two models

**Total number of teeth with DMT prevalence ratio (95% CI)**	**Variables**	**Model 1**	**Model 2**
Intercept		5.59 (4.5-7.0)***	9.1 (4.2-19.7)***
Sex	Female	1.4 (0.99-1.9)	1.2 (0.83-1.6)
PERT prescribed	Yes		0.79 (0.52-1.2)
AZT prescribed	Yes		1.2 (0.85-1.8)
On nutritional supplements	Yes		0.66 (0.42-1.1)
PpFEV1			0.99 (0.98-1.0)
Debris total			1.0 (0.98-1.06)
Key: p <0.1; *p <0.05; **p <0.01; ***p <0.001 Note = Model 1: CF status and sex as the independent variables; Model 2: Model 1 plus disease-specific factors of PERT, AZT, ONS, ppFEV1 and debris total			

## Discussion

This study is the largest global study assessing caries levels in adults with CF. It forms part of a wider study which assessed periodontal health,^21^ oral hygiene status,^21^ enamel defects prevalence,^9^ and caries, which this paper focuses solely on. In this study, PwCF had significantly more caries compared to people without CF. Established risk factors for caries include regular intake of a high sugar-diet and poor oral hygiene. Additional risk factors identified in PwCF included altered or reduced salivary function, higher counts of cariogenic bacteria, GERD and a higher prevalence of developmental defects of enamel.^7^ Data from the self-reported questionnaire demonstrated a trend that PwCF consume more meals and snacks daily compared to the control group. It also highlighted a disparity in dental attendance and brushing habits between the two groups. PwCF brushed less frequently than non-CF individuals and attended the dentist less regularly for check-ups, presenting only if they needed treatment or were in pain. Routine dental attendance is associated with better oral health outcomes and fewer missing teeth.^22^ Across both groups, individuals with second- and third-level education had lower levels of caries. Education can be an indication of social and economic status and can determine an individual's access to dental services.

This study found no differences when variables specific to CF, such as PERT, AZT, ONS and ppFEV1, were analysed. This was interesting, as ONS can contain large amounts of fermentable carbohydrates.^23^ Long-term low dose use of AZT has beneficial effects on the severity of lung disease in PwCF. In this study, AZT did not have any effect on caries levels. This was of interest to the authors, as previous studies reported lower levels of caries in children taking long-term antibiotics.^12^ One plausible reason is that AZT does not have anti-cariogenic effects, whereas childhood antibiotics reduce cariogenic bacteria.

Our findings are comparable to those reported by Pawlaczyk-Kamieńska *et al.*^24^ and Hildebrandt *et al.,*^25^ who conducted similar studies in Poland. All three studies share similar methodology, caries measurement indices and sample populations. Similar to our study, both studies reported more caries (DT) in adults with CF. Our study reported a lower mean number of carious teeth (DT) than that reported by Hildebrandt *et al.*^25^ (0.99 ± 1.825 standard deviation [SD] versus 4.28 ± 3.20, respectively). One plausible reason for this may be the study location. Unlike Ireland, Poland does not have a national public water fluoridation scheme. Over 73% of the Irish population have access to fluoridated water, which has been set at between 0.6-0.8 mg/l.^26^ In Poland, most localities have a fluoride content of below 0.5 mg/l.^27^ Therefore, access to fluoridated water may account for lower levels of carious teeth (DT) in this study.

Caries levels in Ireland have decreased since the introduction of public water fluoridation in 1964. It is interesting to note that the mean DMFT of both groups in this study (6.52 ± 5.004 SD and 5.33 ± 3.956 SD) is lower than the national mean outlined in the ‘Oral health of Irish adults' study (4.9 with a 0.3 standard error for people aged 16-24 years, and 15.0 with a standard error of 0.5 for people aged 35-44 years).^28^ This is the most recent comparable national data. The national survey was conducted in 2000-2002. Within this near 20-year period, oral health in Ireland may have improved because of continued water fluoridation, access to dental care and greater levels of patient awareness. These differences may account for the lower DMFT reported in this current study when compared to national data.

As part of a wider study, both the study and control groups were examined for the presence of developmental enamel defects (DDE). This data are reported in a separate paper.^9^ DDE are defects in the quality and/or quantity of enamel. While they do not cause dental caries, their presence can predispose a tooth to dental caries.^29^ In this study, 64% (n = 59) of study participants had at least one enamel defect compared to just 30% (n = 28) of control group participants.^9^ A higher number of enamel defects may increase the risk of caries.

A limitation of the study is the small sample size. Before recruitment, a power calculation was not conducted due to a lack of studies in this area. As this was a single centre study of a rare disease, the objective was to recruit as many participants as possible. With a sample size of n = 92, we recruited just over 55.5% of adults attending this CF unit. Taking a multicentre approach may be beneficial to increase sample size. As previously mentioned, in this study, caries were recorded at cavitation level using the DMF index, and while this allows for easier examiner calibration and reproducibility particularly in field conditions, it underestimates caries, as does a lack of radiographic supplementation. This is a further limitation of the study and use of alternative caries indices, like the ICDAS or CAST indices, may provide more accurate results.^30^ A final limitation of the study is its cross-sectional design; a longitudinal study would allow us to determine caries incidence over time.

## Conclusion

This study provides information regarding caries levels and historic treatment of PwCF. Within this study, PwCF have been identified as having statistically significantly more dental caries compared to their non-CF counterparts. PwCF have more MT and FT compared to people without CF; although, these differences were not significant. The study has identified sociobehavioural characteristics, such as reduced frequency of toothbrushing, poorer dental attendance, and a higher frequency of snacking and grazing, that may account for a disparity in caries levels and treatment levels between the two groups. National oral health policy in Ireland has two key goals: to ‘provide the supports to enable every individual to achieve their personal best oral health' and to ‘reduce oral health inequalities across the population, by enabling vulnerable groups to access oral healthcare and improve their oral health'.^31^ At present, dentists do not form part of the multidisciplinary health team involved in the care of PwCF; however, considering the findings of this study, it may be beneficial for dentists to join this team so that PwCF can avail of access to dental care. At the very least, PwCF should be encouraged to attend their dentist regularly.

## Data Availability

The data that support the findings of this study are available from the corresponding author upon reasonable request.
